# Supplementary figures and images for: Mitochondrial proline catabolism activates Ras1/cAMP/PKA-induced filamentation in Candida albicans
Source: PLoS Genet. 2019 Feb 11;15(2):e1007976. doi: 10.1371/journal.pgen.1007976 (PMC6386415; doi:10.1371/journal.pgen.1007976)

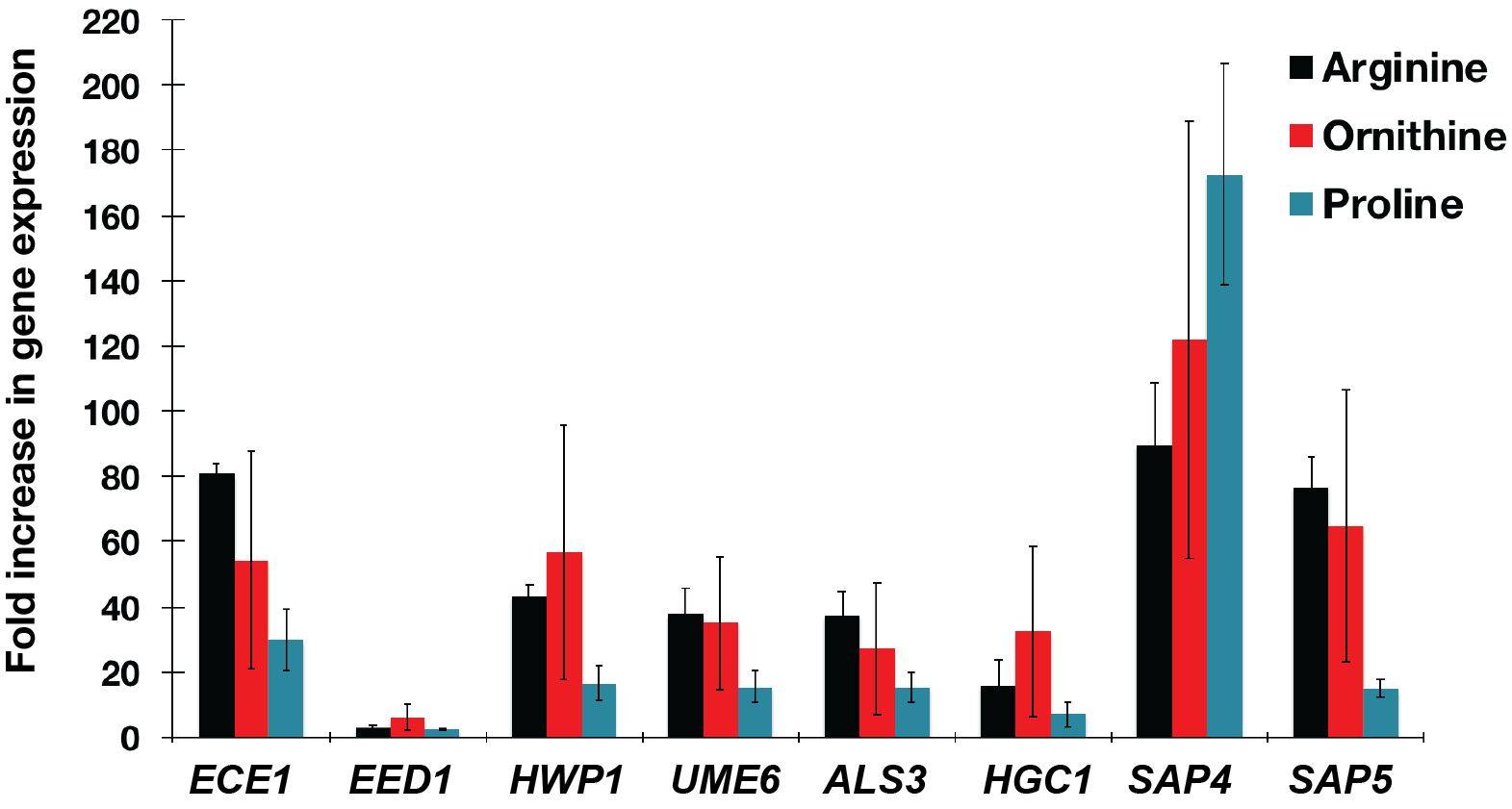

Supplement: S1 Fig — (TIF) [file pgen.1007976.s001.tif]

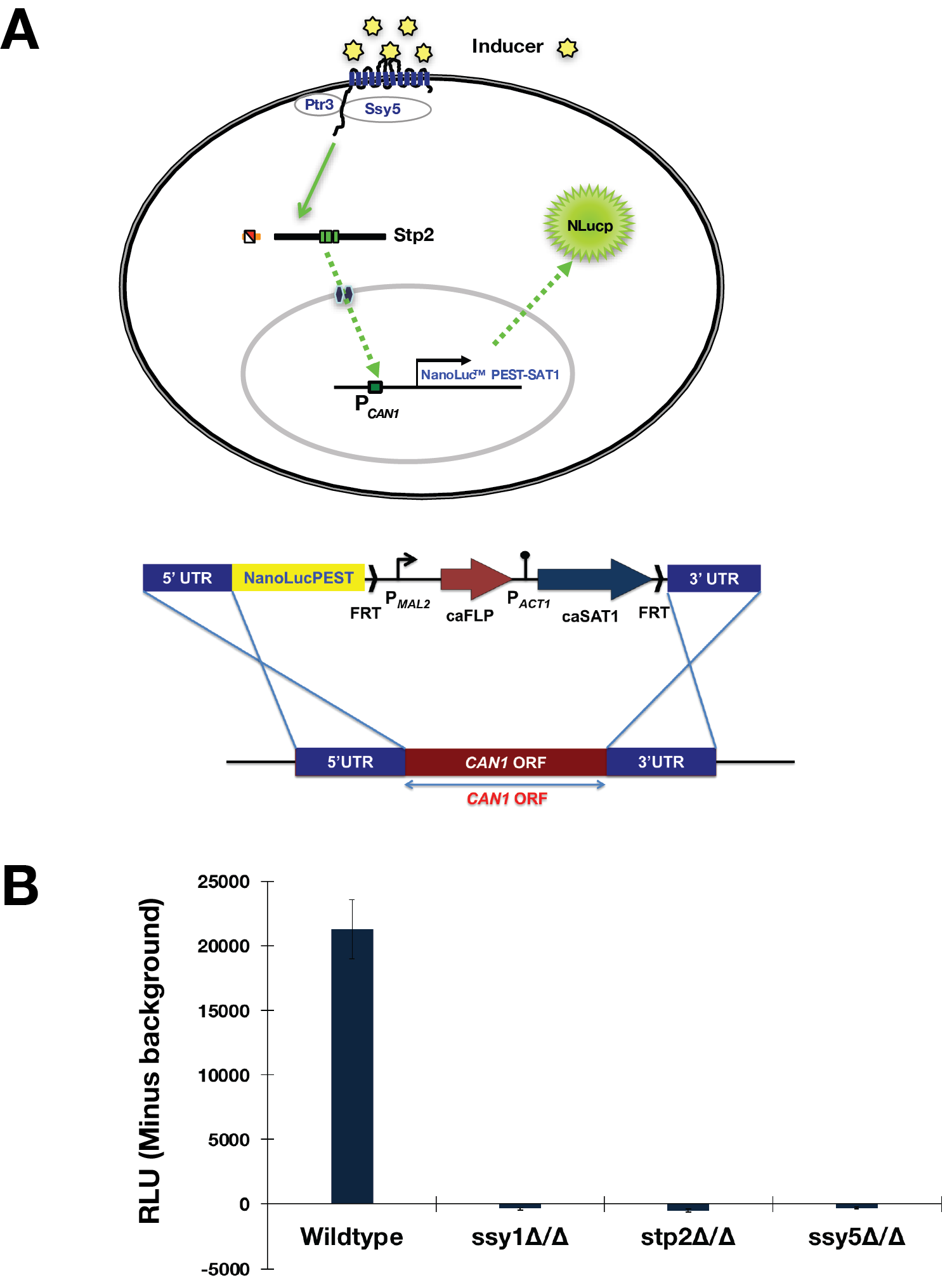

Supplement: S2 Fig — A. The CAN1 promoter (PCAN1) is responsive to extracellular amino acids. B. PCAN1-NanoLucPEST expression is strictly dependent on a functional SPS sensor. (TIF) [file pgen.1007976.s002.tif]

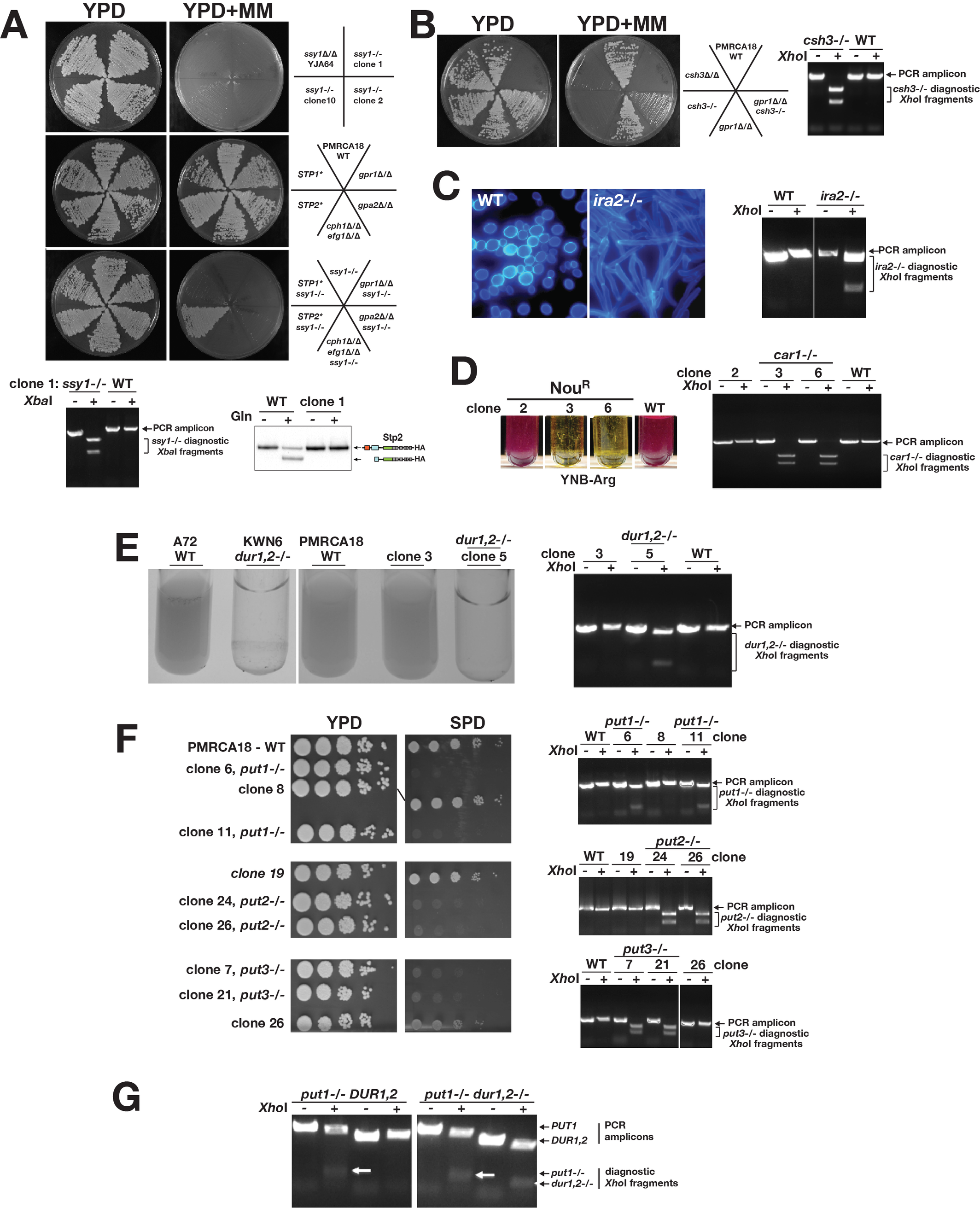

Supplement: S3 Fig — Verification of ssy1-/- strains. B. Verification of csh3-/- strains. C. Verification of ira2-/- strains. D. Verification of car1-/- strains. E. Verification of dur1,2-/- strains. F. Verification of put1-/-, put2-/- and put3-/- strains. G. Verification of the put1-/- dur1,2-/- double mutant strain. (TIF) [file pgen.1007976.s003.tif]

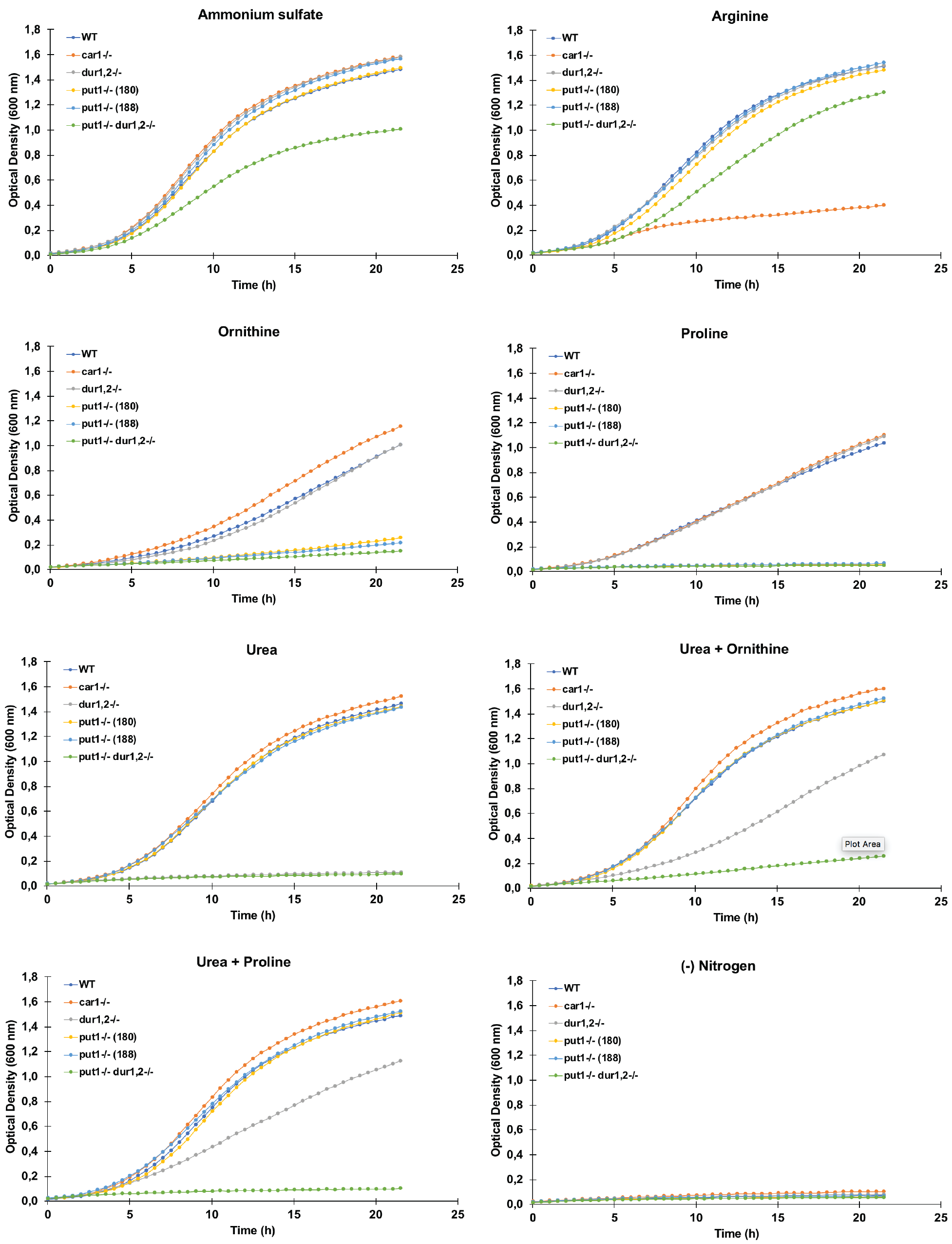

Supplement: S4 Fig — (TIF) [file pgen.1007976.s004.tif]

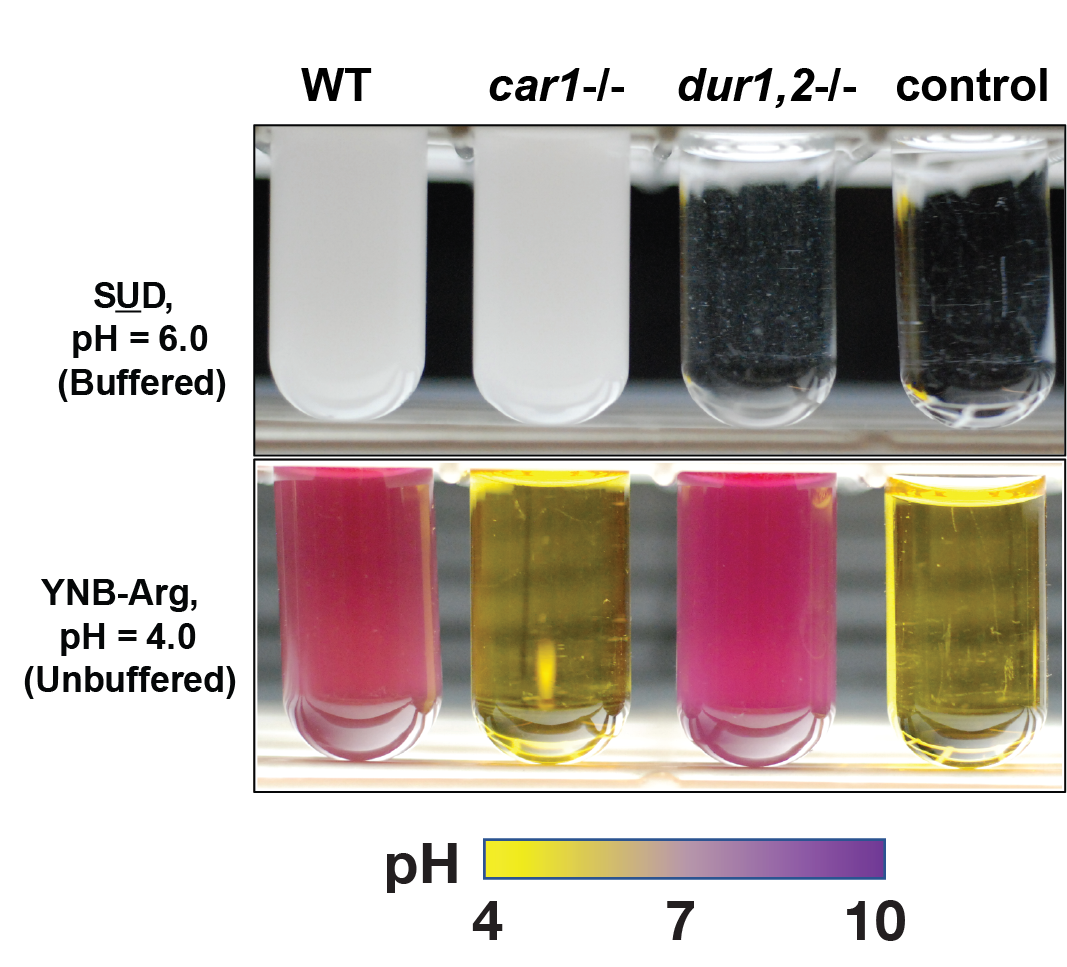

Supplement: S5 Fig — (TIF) [file pgen.1007976.s005.tif]

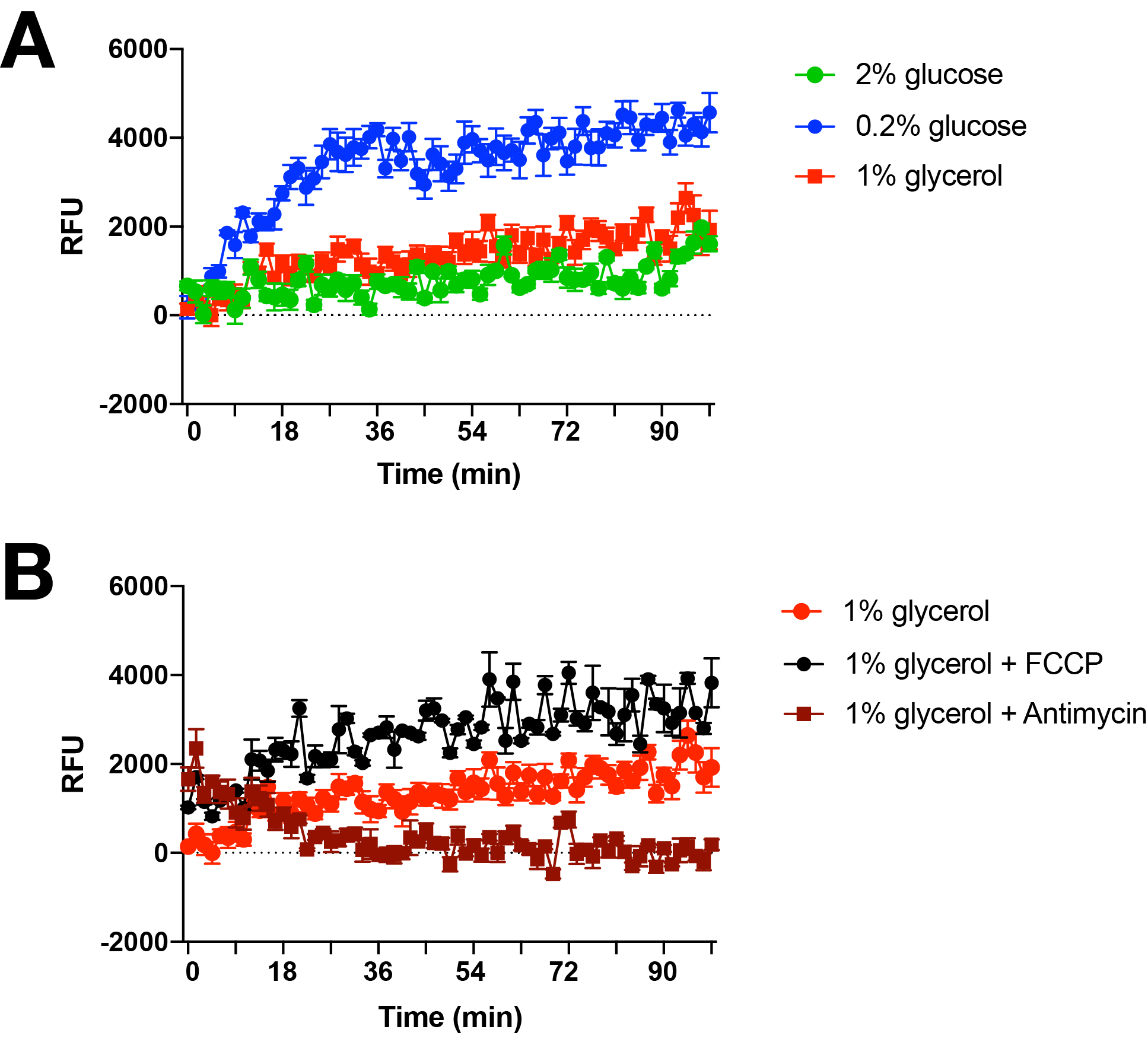

Supplement: S6 Fig — A. Oxygen consumption of C. albicans wildtype cells (PMRCA18) grown in synthetic proline medium containing 2% glucose (SPD), 0.2% glucose (SPD0.2%) or 1% glycerol (SPG). B. Inhibitors of mitochondrial oxidative phosphorylation used to control assessment of oxygen consumption. (TIF) [file pgen.1007976.s006.tif]

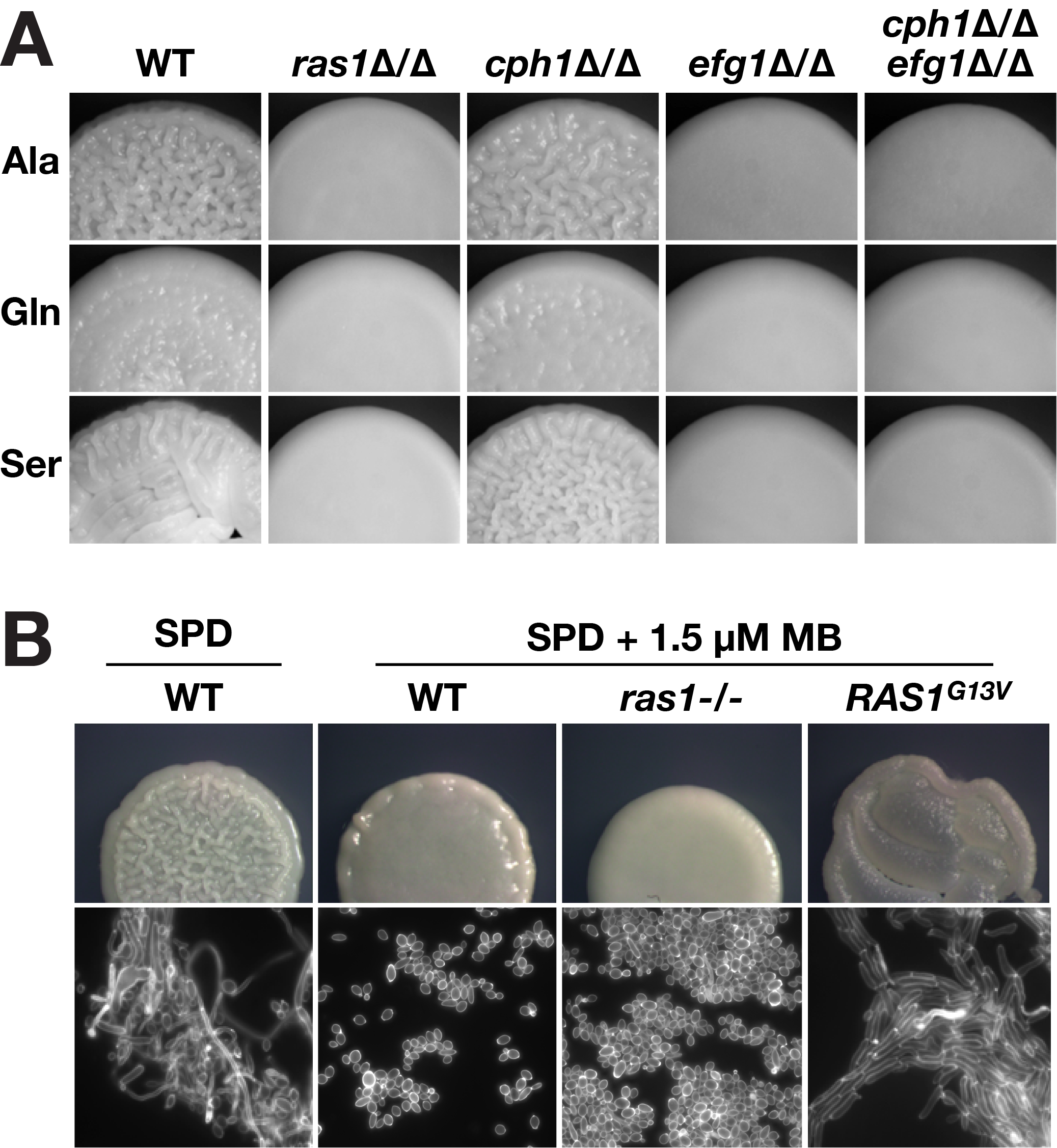

Supplement: S7 Fig — A. Alanine, Glutamine or Serine induce hyphal growth in a Ras1- and Efg1-dependent manner. B. Hyperactive Ras1 (RAS1G13V) bypasses methylene blue inhibition of hyphal growth. (TIF) [file pgen.1007976.s007.tif]
