# Supplementary material for: Mitochondrial proline catabolism activates Ras1/cAMP/PKA-induced filamentation in Candida albicans
Source: PLoS Genet. 2019 Feb 11;15(2):e1007976. doi: 10.1371/journal.pgen.1007976 (PMC6386415; doi:10.1371/journal.pgen.1007976)
Supplement: S1 Text — (DOCX) [file pgen.1007976.s008.docx]

**S1 Text**

**S1 Table. Strains used in this study.**

| - **Strains** | - **Genotype** | - **Reference** |
| --- | --- | --- |
| - **CAI4-derived strains** | |  |
| - CAI4 | *ura3*Δ::*imm434/ura3*Δ::*imm434* | - 106 |
| - CDH107 | *ura3*Δ::*imm434/ura3*Δ::*imm434 ras1*Δ::*hisG/ras1*Δ::*hisG-URA3-hisG* | - 23 |
| - CFG001 | - *ura3*Δ::*imm434/ ura3*Δ::*URA3 CAN1/can1::P_CAN1_-Nanoluc-PEST* | - This work |
| - CFG008 | - *ura3*Δ::*imm434/ ura3*Δ::*URA3 ssy1*Δ::*FRT*/*ssy1*Δ::*FRT CAN1/can1::P_CAN1_-NanolucPEST* | - This work |
| - CFG010 | - *ura3*Δ::*imm434/ ura3*Δ::*URA3 ssy5*Δ:*FRT*/*ssy5*Δ::*FRT CAN1/can1::P_CAN1_-NanolucPEST* | - This work |
| - CFG012 | - *ura3*Δ::*imm434/ura3*Δ::*imm434 stp2*Δ6::*dpl200*-*URA3*/*stp2*Δ2::Ca*NAT1*/*stp2*Δ5::*MPA CAN1/can1::P_CAN1_-NanolucPEST* | - This work |
| - CFG048 | - *ura3*Δ::*imm434/ura3*Δ::*imm434 cph1*Δ::*hisG/cph1*Δ::*hisG efg1*Δ::*hisG/efg1*Δ::*hisG-URA3-hisG ENO1/eno1::P_ENO1_-CC9-pFS017 csh3*- /*csh3-* | - This work |
| - CFG049 | - *ura3*Δ::*imm434/ura3*Δ::*imm434 cph1*Δ::*hisG/cph1*Δ::*hisG efg1*Δ::*hisG/efg1*Δ::*hisG-URA3-hisG ENO1/ eno1::P_ENO1_-CC9-pFS013 ssy1*-/*ssy1*- | - This work |
| - CFG056 | - *ura3*Δ::*imm434/ ura3*Δ::*URA3 ENO1/ eno1::P_ENO1_-CC9-pFS013 ssy1*-/*ssy1*- | - This work |
| - CFG059 | - *ura3*Δ::*imm434/ura3*Δ::*imm434 gpr1*Δ::*hisG/gpr1*Δ::*hisG-URA3-hisG ENO1/eno1::P_ENO1_-CC9-pFS017 csh3*- /*csh3-* | - This work |
| - CFG060 | - *ura3*Δ::*imm434/ura3*Δ::*imm434 gpr1*Δ::*hisG/gpr1*Δ::*hisG-URA3-hisG ENO1/ eno1::P_ENO1_-CC9-pFS013 ssy1*-/*ssy1*- | - This work |
| - CFG064 | - *ura3*Δ::*imm434/ura3*Δ::*imm43 gpa2*Δ::*hisG/gpa2*Δ::*hisG-URA3-hisG ENO1/ eno1::P_ENO1_-CC9-pFS013 ssy1*-/*ssy1*- | - This work |
| - CFG072 | - *ura3*Δ::*imm434/ ura3*Δ::*URA3 ENO1/eno1::P_ENO1_-CC9-pFS017 csh3*- /*csh3-* | - This work |
| - CFG073 | - *ura3*Δ::*imm434/ura3*Δ::*imm434 STP2/STP2::STP2*-URA3 ENO1/ eno1::P_ENO1_-CC9-pFS013 ssy1*-/*ssy1*- | - This work |
| - CFG077 | - *ura3*Δ::*imm434/ ura3*Δ::*URA3 ENO1/ eno1::P_ENO1_-CC9-pFS024 car1*- /*car1*- | - This work |
| - CFG078 | - *ura3*Δ::*imm434/ura3*Δ::*imm434 STP1/STP1::STP1*-URA3 ENO1/eno1::P_ENO1_-CC9-pFS013 ssy1*-/*ssy1*- | - This work |
| - CFG082 | - *ura3*Δ::*imm434/ ura3*Δ::*URA3 ENO1/eno1::P_ENO1_-CC9-pFS028 ira2*- /*ira2*- | - This work |
| - CFG083 | - *ura3*Δ::*imm434/ura3*Δ::*imm434 STP2/STP2/STP2::STP2-6xHA-URA3 ENO1/eno1::P_ENO1_-CC9-pFS013 ssy1*-/*ssy1*- | - This work |
| - CFG091 | - *ura3*Δ::*imm434/ ura3*Δ::*URA3 ENO1/eno1::P_ENO1_-CC9-pFS039 dur1,2*- /*dur1,2*- | - This work |
| - CFG122 | - *ura3*Δ::*imm434/ ura3*Δ::*URA3 ENO1/eno1::P_ENO1_-CC9-pFS080 put1*- /*put1*- | - This work |
| - CFG128 | - *ura3*Δ::*imm434/ ura3*Δ::*URA3 ENO1/eno1::P_ENO1_-CC9-pFS083 put2-* /*put2*- | - This work |
| - CFG130 | - *ura3*Δ::*imm434/ ura3*Δ::*URA3 ENO1/eno1::P_ENO1_-CC9-pFS084 put3-* /*put3*- | - This work |
| - CFG141 | - *ura3*Δ::*imm434/ ura3*Δ::*URA3 NEUT5L::P_ENO1_-CC9-pFS088 put1-* /*put1*- | - This work |
| - CFG155 | - *ura3*Δ::*imm434/ ura3*Δ::*URA3 NEUT5L::FRT put1-* /*put1*- | - This work |
| - CFG158 | - *ura3*Δ::*imm434/ ura3*Δ::*URA3 NEUT5L::FRT put1-* /*put1*-  *ENO1/eno1::P_ENO1_-CC9-pFS039 dur1,2*- /*dur1,2*- | - This work |
| - CR216 | - *ura3*Δ::*imm434/ura3*Δ::*imm434 cdc35*Δ::*hisG/cdc35*Δ::*hisG-URA3-hisG* | - 28 |
| HLC52 | - *ura3*Δ::*imm434/ura3*Δ::*imm434 efg1*Δ::*hisG/efg1*Δ::*hisG-URA3-hisG* | - 8 |
| - HLC54 | - *ura3*Δ::*imm434/ura3*Δ::*imm434 cph1*Δ::*hisG/cph1*Δ::*hisG efg1*Δ::*hisG/efg1*Δ::*hisG-URA3-hisG* | - 8 |
| - JKC19 | - *ura3*Δ::*imm434/ura3*Δ::*imm434 cph1*Δ::*hisG/cph1*Δ::*hisG-URA3-hisG* | - 42 |
| - LDR8 | - *ura3*Δ::*imm434/ura3*Δ::*imm434 gpr1*Δ::*hisG/gpr1*Δ::*hisG-URA3-hisG* | - 33 |
| - NM6 | - *ura3*Δ::*imm434/ura3*Δ::*imm434 gpa2*Δ::*hisG/gpa2*Δ::*hisG-URA3-hisG* | - 33 |
| - PMRCA12 | - *ura3*Δ::*imm434/ ura3*Δ::*URA3 csh3*Δ3/*csh3*Δ3 | - 37 |
| - PMRCA18 | - *ura3*Δ::*imm434/ ura3*Δ::*URA3* | - 37 |
| - PMRCA23 | - *ura3*Δ::*imm434/ ura3*Δ::*imm434 STP1/STP1::STP1*-URA3* | - 36 |
| - PMRCA44 | - *ura3*Δ::*imm434/ ura3*Δ::*imm434 STP2/STP2/STP2::STP2*-URA3* | - 36 |
| - PMRCA48 | - *ura3*Δ::*imm434/ ura3*Δ::*imm434 STP2/STP2/STP2::STP2-6xHA-URA3* | - 36 |
| - PMRCA57 | - *ura3*Δ::*imm434/ura3*Δ::*imm434 stp2*Δ6::*dpl200*-*URA3*/*stp2*Δ2::Ca*NAT1*/*stp2*Δ5::*MPA* | - 36 |
| - PMRCA59 | - *ura3*Δ::*imm434/ ura3*Δ::*URA3 stp1*Δ1/*stp1*Δ1 | - 36 |
| - PMRCA94 | - *ura3*Δ::*imm434/ ura3*Δ::*URA3 stp1*Δ1/*stp1*Δ1 *stp2*Δ6::*dpl200*/*stp2*Δ2::Ca*NAT1*/*stp2*Δ5::*MPA* | - 36 |
| - YJA53 | - *ura3*Δ::*imm434/ ura3*Δ::*URA3 ssy5*Δ:*FRT*/*ssy5*Δ::*FRT* | - 95 |
| - YJA59 | - *ura3*Δ::*imm434/ ura3*Δ::*URA3 CAN1/CAN1-GFP* | - 95 |
| - YJA64 | - *ura3*Δ::*imm434/ ura3*Δ::*URA3 ssy1*Δ::*FRT*/*ssy1*Δ::*FRT* | - 95 |
| **SC5314-derived strains** | |  |
| SC5314 | Prototrophic wild type |  |
| CASJ041 | - *cph1*Δ::*FRT/ cph1*Δ::*FRT efg1*Δ::*FRT/ efg1*Δ::*FRT* | - 107 |
| CFG139 | - *NEUT5L::P_ENO1_-CC9-pFS088 put1-* /*put1*- | - This work |
| - CFG143 | - *ENO1/eno1::P_ENO1_-CC9-pFS083 put2-* /*put2*- | - This work |
| - CFG146 | - *NEUT5L::P_ENO1_-CC9-pFS090 put3-* /*put3*- | - This work |
| - CFG149 | - *ENO1/eno1::P_ENO1_-CC9-pFS080 put1*- /*put1*- | - This work |
| - CFG150 | - *ENO1/eno1::P_ENO1_-CC9-pFS084 put3-* /*put3*- | - This work |
| - CFG155 | - *NEUT5L::FRT put1-* /*put1*- | - This work |
| - CFG159 | - *NEUT5L::FRT put1-* /*put1*-  *ENO1/eno1::P_ENO1_-CC9-pFS083 put2-* /*put2*- | - This work |
| - CFG181 | - *ENO1/eno1::P_ENO1_-CC9-pV1093 NEUT5L::pV1524* | - This work |
| - CFG182 | - *NEUT5L::pV1524* | - This work |
| - CFG183 | - *gat1::FRT/gat1::FRT gln3::FRT/gln3::FRT PUT2/PUT2-HA-NAT* | - This work |
| - CFG185 | - *PUT2/PUT2-HA-NAT* | - This work |
| - CFG207 | - *NEUT5L::P_ENO1_-CC9-pFS092 put2-* /*put2*- | - This work |
| CFG240 | - *NEUT5/neut5::FRT put1-/- ADH1/adh1::P_ADH1_-RFP-NAT* | - This work |
| CFG246 | - *ENO1/eno1::CC9-pFS039 dur1,2-/ dur1,2-* | - This work |
| CFG267 | - *ENO1/eno1::CC9-pV1121 RAS1^G13V^/ RAS1^G13V^* | - This work |
| CFG271 | - *ENO1/eno1::CC9-pV1121 ras1-/-* | - This work |
| DHCA216 | - *efg1*Δ::*FRT/ efg1*Δ::*FRT* | - 107 |
| SCADH1G4A | - *ADH1/adh1::P_ADH1_-GFP-NAT* | - 108 |
| - Δgln3GAT1M4A | - *gat1::FRT/gat1::FRT gln3::FRT/gln3::FRT* | - 108 |
| - **A72-derived strains** | |  |
| A72 | - Protrophic wild type | - 32 |
| KWN6 | - *dur1,2*Δ::FRT/ *dur1,2*Δ::FRT | - 32 |
