# Supplementary material for: Mitochondrial proline catabolism activates Ras1/cAMP/PKA-induced filamentation in Candida albicans
Source: PLoS Genet. 2019 Feb 11;15(2):e1007976. doi: 10.1371/journal.pgen.1007976 (PMC6386415; doi:10.1371/journal.pgen.1007976)
Supplement: S2 Text — (DOCX) [file pgen.1007976.s009.docx]

**S2 Text**

**S2 Table. Primers used in this study**

| - **No** | - **Primer Name** | | | - **Sequence** | **Reference** | | |
| --- | --- | --- | --- | --- | --- | --- | --- |
| - **qPCR Primers** | | |  | |  |  |  |
| - 1 | | - FG_qGAP1F | - GAAGTCCATCTACGGGAGAAC | | - This study |  |  |
| - 2 | | - FG_qGAP1R | - CCTATAGATGATCCAATAGCGATC | | - This study |  |  |
| - 3 | | - FG_qGAP2F | - GTACAGTTATGTCCCTTGGG | | - This study |  |  |
| - 4 | | - FG_qGAP2R | - CAGCTTCACCGTAACCACGG | | - This study |  |  |
| - 5 | | - FG_qGAP3F | - CTGCTTCAGAATAGTCCTGAATC | | - This study |  |  |
| - 6 | | - FG_qGAP3R | - GGATGAAGCTGATAATGATGACG | | - This study |  |  |
| - 7 | | - FG_qGAP4F | - CAGTCTGCTTCTACTGCTACTG | | - This study |  |  |
| - 8 | | - FG_qGAP4R | - GAGTTAATTGCGAAGTATCAATAC | | - This study |  |  |
| - 9 | | - FG_qGAP5F | - CCATTGACAGATTTAACATCTCC | | - This study |  |  |
| - 10 | | - FG_qGAP5R | - CATCACGATAACGCATGGCAAG | | - This study |  |  |
| - 11 | | - FG_qGAP6F | - CGAGTGCTACACAACATCGAC | | - This study |  |  |
| - 12 | | - FG_qGAP6R | - GTTGTAATGGAACATTCTGTCC | | - This study |  |  |
| - 13 | | - FG_qACT1For | - CCAACTGGGACGATATGGAA | | - This study |  |  |
| - 14 | | - FG_qACT1Rev | - ATTGGAGCTTCGGTCAACAA | | - This study |  |  |
| - 15 | | - FG_qPMA1For | - GTTGAAAATGCCGATGGTTT | | - This study |  |  |
| - 16 | | - FG_qPMA1Rev | - ACCAGTCATGGCAACCAAGT | | - This study |  |  |
| - 17 | | - ECE1-R1 | - ATCGAAAATGCCAAGAGAG | | - This study |  |  |
| - 18 | | - ECE1-R2 | - AGCATTTTCAATACCGACAG | | - This study |  |  |
| - 19 | | - EED1-R1 | - TAGTGGTAATACCCAACGTG | | - This study |  |  |
| - 20 | | - EED1-R2 | - CTGATATTTGAAATTTTGGAAGCTTTTC | | - This study |  |  |
| - 21 | | - HWP1-R1 | - ATCAGCTCCTGCCACTGAAC | | - This study |  |  |
| - 22 | | - HWP1-R2 | - TGAGTGGAACTGATTCTAATGTAGTTG | | - This study |  |  |
| - 23 | | - UME6-R1 | - TCTACTTCTAATCCAATGGTG | | - This study |  |  |
| - 24 | | - UME6-R2 | - TATCATTACTTGATTTTTTCCGAG | | - This study |  |  |
| - 25 | | - RT ALS3 for | - TCTCGTCCTCATTACACCAACCAT | | - This study |  |  |
| - 26 | | - RT ALS3 rev | - GGGGATTGTAAAGTGGATTCTGTG | | - This study |  |  |
| - 27 | | - 40 SAP4_fwd | - agatattgagcccacagaaatttcc | | - 109 |  |  |
| - 28 | | - 97 SAP4_rev | - ccttgtgcaacagaaccatcg | | - This study |  |  |
| - 29 | | - 98 SAP5_fwd | - cttattccccagcatcttccc | | - This study |  |  |
| - 30 | | - 99 SAP5_rev | - ggaagattgtcgtaatcaaactc | | - This study |  |  |
| - 31 | | - 100 HGC1_forw | - CCAAAATCAATTTCACAAC | | - This study |  |  |
| - 32 | | - 101 HGC1_rev | - GGTTTCAATCAAATTAACC | | - This study |  |  |
| - 33 | | - PUT3 qPCR fwd | - CCAAACTACATGACGAAATAGC | | - This study |  |  |
| - 34 | | - PUT3 qPCR rev | - CCATTTGCTAATGGTGTTTGTG | | - This study |  |  |
| - 35 | | - DUR1,2 fwd | - CTCTCCGTCAAAAGATGCCAC | | - This study |  |  |
| - 36 | | - DUR1,2 rev | - GCCATAATATTAAACGTAAGC | | - This study |  |  |
| - 37 | | - PUT1 fwd#2 | - CCCATGTCAATTATTAAAGCTC | | - This study |  |  |
| - 38 | | - PUT1 rev#2 | - GGTTTCAATTGATTTGGCAG | | - This study |  |  |
| - 39 | | - PUT2 fwd#2 | - GGAACCGTGCTGAATATAGACC | | - This study |  |  |
| - 40 | | - PUT2rev#2 | - GCTGGCAAACCGGCTTCTTCC | | - This study |  |  |
| - 41 | | - RIP1-fwd | - accaccaccaccttatccaa | | - This study |  |  |
| - 42 | | - RIP1-rev | - aatggagctggaccctttct | | - This study |  |  |
|  | | | | |  |  |  |
| - **CRISPR/Cas9 Primers** | | | | |  |  |  |
| 43 | | - sgRNA-CSY1T | - atttgCTACCGCAGCGAAACCAAGGg | | - This study | |  |
| 44 | | - sgRNA-CSY1B | - aaaacCCTTGGTTTCGCTGCGGTAGc | | - This study | |  |
| 45 | | - RT-SSY1Top | - CATCTTTAAACGACAGGATATTTCATGATTTAcaagagagaCTACCGCAGCGAAACCAAGGc**TAG**tctagacaactgg | | - This study | |  |
| - 46 | | - RT-SSY1Bot | - ccaaatccaaatccaaataattttggaaattttcatcttgtcttcCCAGTTGtctaga**CTA**gCCTTGGTTTCGCTGCGGTAG | | - This study | |  |
| - 47 | | - SSY1-VerF | - CTGTCTCCGGGTAAAGAATG | | - This study | |  |
| - 48 | | - SSY1-VerR | - CCAATAAATTCCAAGTGAGGC | | - This study | |  |
| - 49 | | - sgRNA-CSH3T | - atttgTACGTCATTATCAACAATGGg | | - This study | |  |
| - 50 | | - sgRNA-CSH3B | - aaaacCCATTGTTGATAATGACGTAc | | - This study | |  |
| - 51 | | - RT-CSH3Top | - CTTAACACTTTATGGATTCATGATATCCCTGAAGcattcgatgcctcttTACGTCATTATCAACAATGG**TAA**ctcgagctctcc | | - This study | |  |
| - 52 | | - RT-CSH3Bot | - gatgaaatgtccaattaaacctaataatccaacagcgtgtaatgtataatgaacatacattGGAGAGctcgag**TTA**CCATTgttgataatgacg | | - This study | |  |
| - 53 | | - CSH3-VerF | - GGAGAATGTGGACCATATTCTGCA | | - This study | |  |
| - 54 | | - CSH3-VerR | - TGAGCCCTATTGGTTAACCAG | | - This study | |  |
| - 55 | | - sgRNA-CAR1T | - atttgTCAGGTGGTCAACCAAAAGGg | | - This study | |  |
| - 56 | | - sgRNA-CAR1B | - aaaacCCTTTTGGTTGACCACCTGAc | | - This study | |  |
| - 57 | | - RT-CAR1Top | - CATCCAGACAAAAAAGCTTCTATAATAACAGCACCATTTTCAGGTGGTCAACCAAAAGGT**TAA**TctcgagTAGAAT | | - This study | |  |
| - 58 | | - RT-CAR1Bot | - GATTCAATTTGTTTTTGGAAACCAGCTTTGAGAATATAATCAGGACCCAATTCTActcgagA**TTA**ACCTTTTGGTTGACCACC | | - This study | |  |
| - 59 | | - CAR1-VerF | - CCACAACCATTGGGCATTTAG | | - This study | |  |
| - 60 | | - CAR1-VerR | - GCTAACATTGGGTTAGTCTCC | | - This study | |  |
| - 61 | | - sgRNA-DUR1,2T | - atttgTTCAGCCGGATCTGCCTCGGg | | - This study | |  |
| - 62 | | - sgRNA-DUR1,2B | - aaaacCCGAGGCAGATCCGGCTGAAc | | - This study | |  |
| - 63 | | - RT-DUR1,2Top | - GAATAACTCCAAATACATTCAATCCAAAGTACGTCAGTGGTGGATCTTCAGCCGGATCTGCCTCG**TAG**ctcgagGCTAGAGG | | - This study | |  |
| - 64 | | - RT-DUR1,2Bot | - CTCTTCCTGATCCAGCAGTATCGGTACCTAATGCAATTGGAACAATACCTCTAGCctcgag**CTA**CGAGGCAGATCCG | | - This study | |  |
| - 65 | | - DUR1,2-VerF | - GGTTGGTCAGTAGAAGATTG | | - This study | |  |
| - 66 | | - DUR1,2-VerR | - GCTAGTGCAGACATATCTGC | | - This study | |  |
| - 67 | | - sgRNA-IRA2T | - atttgTAATTATAAGCAAAACCAGGg | | - This study | |  |
| - 68 | | - sgRNA-IRA2B | - aaaacCCTGGTTTTGCTTATAATTAc | | - This study | |  |
| 69 | | - RT-IRA2Top | - CATTTTGATAAATTCAGATAAATACATTAGAATTGTCATCAATAATTATAAGCAAAACCAG**TAGTAA**ActcgagCGGTTCAACAAAA | | - This study | |  |
| 70 | | - RT-IRA2Bot | - CTGTCTGAATTTCTGATAACCCTTTAATTATCTTGACAAAAATTTTGTTGAACCGctcgagT**TTACTA**CTGG | | - This study | |  |
| - 71 | | - IRA2-VerF | - CCAATTGATACCATCCACTC | | - This study | |  |
| - 72 | | - IRA2-VerR2 | - CCGACAATAACTCTTCTGCTC | | - This study | |  |
| - 73 | | - pCas9-PUT1T | - atttgGTTTATCGTATTTATTGTGGg | | - This study |  |  |
| - 74 | | - pCas9-PUT1B | - aaaacCCACAATAAATACGATAAACc | | - This study |  |  |
| - 75 | | - RT-PUT1Top | - CCATGTCAATTATTAAAGCTCTTGTTTATCGTATTTATTGTGGT**TAA**TctcgagCCATTGATCAAGTTAAAAAAAC | | - This study |  |  |
| - 76 | | - RT-PUT1Bot | - GAAATCATCATATTATTAATACCTCGTTCATGCAATCTTAAGCCAGTTTTTTTAACTTGATCAATGGctcgagA**TTA**ACC | | - This study |  |  |
| - 77 | | - PUT1-VerF | - CATCGGTTATTATTCTTCTTG | | - This study |  |  |
| - 78 | | - PUT1-VerR | - GTTTAACCACTTCCAAATAATC | | - This study |  |  |
| - 79 | | - pCas9-PUT2T | - atttgTACTCGTAATACATTACGTTg | | - This study |  |  |
| - 80 | | - pCas9-PUT2B | - aaaacAACGTAATGTATTACGAGTAc | | - This study |  |  |
| - 81 | | - RT-PUT2Top | - CAGACATACATATTCATTTATAATGTTAAGATCAACTACTCGTAATACATTA**TAA**A**TGA**ctcgagTACTAG | | - This study |  |  |
| - 82 | | - RT-PUT2Bot | - GTGTGACGAATGATACTTGATGAAACTTTAGTATATCTAGTActcgag**TCA**T**TTA**TAATGTATTAC | | - This study |  |  |
| - 83 | | - PUT2-VerF | - CTAGCGGATTAACTATTCGC | | - This study |  |  |
| - 84 | | - PUT2-VerR | - GGATAATGCGGCTGTAGCAG | | - This study |  |  |
| - 85 | | - pCas9-PUT3T | - atttgGGATTCACAAGAGCCTTCGGg | | - This study |  |  |
| - 86 | | - pCas9-PUT3B | - aaaacCCGAAGGCTCTTGTGAATCCc | | - This study |  |  |
| - 87 | | - RT-PUT3Top | - CATTCCTTCATTTACTTATATATAATCCGATTCTTGTACAATGGATTCACAAGAGCCT**TAA**ctcgag**TGA**AGAAAATTGC | | - This study |  |  |
| - 88 | | - RT-PUT3Bot | - CAAGTGGAATGGTATCTGAATTAATTAATGCATTTGCAATTTTCT**TCA**ctcgag**TTA**AGGCTCTTG | | - This study |  |  |
| - 89 | | - PUT3-VerF | - CGTGCATTACTTCATGTAATC | | - This study |  |  |
| - 90 | | - PUT3-VerR | - GGACGAAGGTATTGTTTGAGG | | - This study |  |  |
| - 91 | | FS95 | - ggcatagctgaaacttcggc | | - This study |  |  |
| - 92 | | RT-RAS1Top143 | - ATATCCACACATATACATACCATGTTGAGAGAATATAAATTAGTTGTTGTTGGAGGTGtT | | - 91 |  |  |
| - 93 | | RT-RAS1Bot143 | - AATCAATTGAATGGTTAAAGCGGATTTACCAACACCAaCACCTCCAACAACAACTAATTT | | - 91 |  |  |
| - 94 | | RT-RAS1TopTAA | - ATATCCACACATATACATACCATGTTGAGAGAATATAAATTAGTTGTTGTTGGAGGTtaaGA | | - 91 |  |  |
| - 95 | | RT-RAS1BotTAA | - AATCAATTGAATGGTTAAAGCGGATTTACCAACACCGAATTCTTAACCTCCAACAACAAC | | - 91 |  |  |
| 96 | | RAS1-VerF | - TCAATTGACTAGATATAAACTCTTC | | - 91 |  |  |
| - 97 | | RAS1-VerR | - TCCATCTTCATAACTAACTTGTCTT | | - 91 |  |  |
| - 98 | | sgRNA-RAS1T | - atttgAAATTAGTTGTTGTTGGAGGg | | - 91 |  |  |
| 99 | | sgRNA-RAS1B | - aaaacCCTCCAACAACAACTAATTTc | | - 91 |  |  |
| - **NanoLuc cloning primers** | | |  | |  |  |  |
| - 100 | | - CAN1NCRUpF_KI | - CACGTAggtaccCATAACTCAATTCATTGATCTCAC | | - This study |  |  |
| - 101 | | - CAN1NCRUpR_XI | - CAATTActcgagGAGGAGATATATGATATATTTAGAG | | - This study |  |  |
| 102 | | - NanolucF_XhoI | - CAAATTctcgagCAAGTAAATAACAGATAATATG | | - This study |  |  |
| 103 | | - Nanoluc_BamHI | - CATACAggatccGGTATTCTGGGCCTCCATGTC | | - This study |  |  |
| 104 | | - CAN1NCRDwF_XbI | - CATTAAtctagaCTGGGCTATTGTTGCATAAACG | | - This study |  |  |
| 105 | | - CAN1NCRDwR_NI | - CATATTgcggccgcGTGTTCCAGAGCATCGATTATGG | | - This study |  |  |
| 106 | | - CAN1VerifDwnR | - CTAATCATCAAGATCCAACAGG | | - This study |  |  |
| 107 | | - CAN1FOverlap | - GCCATCGCATCTAACCAACCATG | | - This study |  |  |
| - **Epitope-tagging primers** | | |  | |  |  |  |
| 108 | | - C-HATagPUT2F | - CTGGTAGTGGTAACATTTTATCCAGATTTGTTTCTATTAGAAACATTAAAGAAAACTTTTACGAATTGACTGATTTCAAATATCCATCCAATTATCAAaacatcttttacccatacgatg | | - This study |  |  |
| 109 | | - C-HATagPUT2R | - GGAAACAACATGAACACCTTATGTAAGAAAACCTCTTCTTAATATAAATATTTACATTCACACATTAACTATATAAAGTAATAACTAATCTCGTTTCTCgcaggttaacctggcttatcg | | - This study |  |  |
| 110 | | - FS244_HAfor | - CTAGCGGATTAACTATTCGC | | - This study |  |  |
| 111 | | - FS340_HArev | - cgtcatatggataggatcctg | | - This study |  |  |
| 112 | | - 5'ADH1test | - ACAATATTTGATAGAGAC | | - This study |  |  |
| 113 | | - 3'ADH1test | - TTGAATCTACGAGACTC | | - This study |  |  |
