# Supplementary material for: Mitochondrial proline catabolism activates Ras1/cAMP/PKA-induced filamentation in Candida albicans
Source: PLoS Genet. 2019 Feb 11;15(2):e1007976. doi: 10.1371/journal.pgen.1007976 (PMC6386415; doi:10.1371/journal.pgen.1007976)
Supplement: S3 Text — (DOCX) [file pgen.1007976.s010.docx]

**S3 Text**

**Supporting Figures - Legends**

**S1 Fig. Hyphae-specific gene (*HSG*) expression in *C. albicans* grown in the presence of inducing amino acids.** Total RNA was extracted from pooled 24 h-old colonies of wildtype strain (PMRCA18) scraped from the surface of the indicated SXD plates grown at 37 °C. *HSG* expression was monitored by RT-qPCR using the primers listed in Table S2 and normalized to the *ACT1* housekeeping gene. Expression levels of *HSG* in inducing amino acids were compared to aspartic acid that serve as reference (yeast) expression. The results are the average of 3 biological replicates (ave. ± CI, 95% CL).

**S2 Fig.** **NanoLuc luciferase assay for analysis of Stp2 target gene expression.** **A.** The *CAN1* promoter (*P_CAN1_*) is responsive to extracellular amino acids. The plasma membrane-localized SPS sensor proteolytically activates transcription factor Stp2, which binds and induces the *P_CAN1_* promoter. Schematic diagram of the Nlucp SAT1-Flipper Cassette used to place the *NanoLucPEST* (*Nlucp*) under the control of the *CAN1* promoter. **B.** *P_CAN1_*‑*NanoLucPEST* expression is strictly dependent on a functional SPS sensor. Log phase cells in SD were induced with 50 μM of the indicated amino acids for 2 h at 30 °C and then analyzed for luciferase signal as described in the Materials and Methods. Results shown are the average of five biological replicates with readings from ddH_2_O-spiked cells serving as background control (ave. ± CI, 95% CL). Strains: *CAN1/P_CAN1_-Nlucp* (wildtype, WT; CFG001) and SPS-deficient mutants, *ssy1*Δ/Δ *CAN1/P_CAN1_-Nlucp* (CFG008), *stp2*Δ/Δ *CAN1/P_CAN1_-Nlucp* (CFG012)and *ssy5*Δ/Δ *CAN1/P_CAN1_-Nlucp* (CFG010).

**S3 Fig.** **Phenotypic characterization of CRISPR/Cas9-mediated gene inactivation in *C. albicans*.** Strains of the indicated genotypes were transformed with purified *Kpn*I and *Sac*I-digested plasmids harboring gene-specific sgRNA and the corresponding repair template (RT). Nou^R^ transformants, identified by plating cells on YPD-Nou plates, were pre-screened according to the expected phenotype. **A.** Verification of *ssy1*-/- strains. Nou^R^ colonies were screened to identify MM hypersensitive (HS) clones (YPD+MM), a phenotypic characteristic of SPS-sensing pathway inactivation (upper panel; strains *ssy1*Δ/Δ (YJA64), PMRCA18 derived *ssy1-/-* clones 1, 2 and 10). Clone 1 was designated CFG056. Growth of *ssy1*-/- (CFG056), *gpr1*Δ/Δ *ssy1*-/- (CFG060), *gpa2*Δ/Δ *ssy1*-/- (CFG064), *cph1*Δ/Δ *efg1*Δ/Δ *ssy1*-/- (CFG049), *STP2** *ssy1*-/- (CFG073) and *STP1** *ssy1*-/- (CFG078) (lower panel) and their respective parental strains *SSY1* (PMRCA18), *gpr1*Δ/Δ (LDR8), *gpa2*Δ/Δ (NM6), *cph1*Δ/Δ *efg1*Δ/Δ (HLC54), *STP2** (PMRCA44) and *STP1** (PMRCA23) (middle panel). DNA fragments carrying the *SSY1* locus were amplified by PCR using genomic DNA from WT (PMRCA18) and Nou^R^ MM^HS^ (clone 1, CFG056) as template; the PCR amplicon was restricted with *Xba*I. Cleavage verifies that Cas9 cut at the desired genomic locus, and that the cut was repaired using the co-transformed rescue template (*ssy1-/-*). Immunoblot analysis of Stp2 processing (bottom right panel). The lack of Stp2 processing confirms loss of Ssy1 function; strains *STP2*/*STP2-6xHA* (WT; PMRCA23), *STP2*/*STP2-6xHA ssy1*-/- (CFG083)*.* **B.** Verification of *csh3*-/- strains. Nou^R^ colonies were prescreened as in (**A**); growth of strains WT (PMRCA18), *gpr1*Δ/Δ *csh3*-/- (CFG059), *gpr1*Δ/Δ (LDR8), *csh3*-/- (CFG072) and *csh3*Δ/Δ (PMRCA12) (left panels); and PCR and restriction analysis of amplified *CSH3* fragments, *Xho*I cleavage is diagnostic of *csh3-/-*. **C.** Verification of *ira2*-/- strains. Nou^R^ colonies were screened by microscopy; WT (PMRCA18) and *ira2*-/- (CFG082) cells grown in liquid YPD at 30 °C and stained with calcofluor (left panels). A colonies comprised of cells exhibiting constitutive filamentous growth was chosen and inactivation of *IRA2* was verified by PCR and restriction by *Xho*I (right panels). **D.** Verification of *car1*-/- strains**.** Nou^R^ transformants were grown in YNB+Arg medium (pH = 4.0, unbuffered) containing bromocresol purple (BCP) as indicator; clones 3 and 6 lacking the inability to utilize arginine and to alkalinize the medium were analyzed by PCR and restriction by *Xho*I (right panels); strains WT (PMRCA18), *car1*-/- (clone 6, CFG077). **E.** Verification of *dur1,2*-/- strains. Nou^R^ transformants were pre-screened for growth in SUD medium. Clone 5, lacking the ability to utilize urea as sole nitrogen source, was analyzed by PCR and the *dur1,2*-/- mutation was confirmed by *Xho*I restriction. Strains A72 and KWN6 (generous gift from Kenneth Nickerson and Ruvini Pathirana) were used as reference in the growth-based assay; strains WT (A72), *dur1,2*Δ/Δ (KWN6; A72-derivative), WT (PMRCA18), Nou^R^ false positive (clone 3) and *dur1,2*-/- (clone 5, CFG091; PMRCA18-derivative). **F.** Verification of *put1*-/-, *put2*-/- and *put3*-/- strains. Nou^R^ transformants were pre-screened in SPD medium with proline as sole nitrogen source (left panels). PCR analysis with *Xho*I restriction was used to confirm *put1-/-*, *put2-/-* and *put3-/-* mutations. Strains: WT (PMRCA18), *put1‑*/- (clone 6, CFG122), *put2*-/- (clone24, CFG128), *put3*-/- (clone 7, CFG130). **G.** Verification of the *put1*-/- *dur1,2*-/- double mutant strain. *DUR1,2* in *put1*-/- strain (CFG155) was targeted for inactivation. PCR analysis and *Xho*I restriction confirmed the *dur1,2*-/- mutation (*put1*-/- *dur1,2*-/- (CFG158)).

**S4 Fig. Growth of arginase-pathway mutants in media with different nitrogen sources.** Growth of strains with the indicated genotypes in SXD liquid medium was monitored by optical density (OD_600_) using a BioScreen analyzer (X= Am, Arg, Orn, Pro, Urea, Urea+Orn, Urea+Pro or no Nitrogen). Cells with a starting OD_600_ ≈ 0.05 were grown for > 20 h at 30 °C with constant agitation. Results are averages of three biological replicates. Strains: wildtype (WT; PMRCA18), *car1*-/- (CFG077), *dur1,2*-/- (CFG091), *put1*-/- (180; CFG122), *put1*-/- (188; CFG155), and *put1*-/- *dur1,2* -/- (CFG158).

**S5 Fig. Mutants lacking *DUR1,2* retain the ability to alkalinize the growth medium when using arginine as sole carbon and nitrogen source.** Cells from overnight YPD cultures were washed in PBS and then diluted to OD_600_ ≈ 0.1 in SUD (pH = 6.0) or YNB+Arg+BCP (pH = 4.0, unbuffered) liquid medium (i.e. yellow = acidic; purple = alkaline). Cultures were grown for 24 h at 30 °C under vigorous agitation and photographed. Growth of *dur1,2*-/- (CFG091) was compared to wildtype (WT; PMRCA18) and *car1*-/- (CFG077). Sterile ddH_2_O was used as control.

**S6 Fig. Mitochondrial oxygen consumption is carbon source dependent. A.** Oxygen consumption of *C. albicans* wildtype cells (PMRCA18) grown in synthetic proline medium containing 2% glucose (SPD), 0.2% glucose (SPD_0.2%_) or 1% glycerol (SPG). Cells from log phase YPD cultures were harvested, washed, and then added to each medium at an OD_600_ ≈ 0.3. Oxygen consumption (Relative Fluorescence Units; RFU) was assessed over a 100 min time period as described in Materials and Methods (Extracellular Oxygen Consumption Assay, Abcam, ab197243). **B.** Inhibitors of mitochondrial oxidative phosphorylation used to control assessment of oxygen consumption. FCCP (final conc. 10 μM) and antimycin (final conc. 10 μg/ml) where added to SPG-grown cells as positive and negative metabolic controls, respectively. The uncoupling activity of FCCP increases oxygen consumption while inhibition of Complex III by antimycin effectively blocks oxygen consumption.

**S7 Fig.** **Ras1/cAMP/PKA pathway signaling and Efg1-dependent transcription are required for amino acid-induced morphogenesis. A.** Alanine, Glutamine or Serine induce hyphal growth in a Ras1- and Efg1-dependent manner. Cells from overnight precultures of wildtype (WT; PMRCA18), *ras1*Δ/Δ (CDH107), *cph1*Δ/Δ (JKC19), *efg1*Δ/Δ (HLC52), and *cph1*Δ/Δ *efg1*Δ/Δ (HLC54) in YPD were harvested, washed, and resuspended to an OD_600_ ≈ 8.0. Ten μl aliquots were spotted on the indicated SXD plate (X = Alanine, Glutamine or Serine). The plates were incubated at 37 °C for 48 h and photographed. **B.** Hyperactive Ras1 (*RAS1^G13V^*) bypasses methylene blue inhibition of hyphal growth. Wildtype (WT; SC5314), *ras1-/-* (CFG271) and *RAS1^G13V^/RAS1^G13V^* (CFG267) were precultured, harvested and resuspended as in **A.** Ten μl aliquots were spotted on SPD supplemented with 1.5 μM of methylene blue (MB). The plates were incubated for 24 h at 37 °C and the resulting colonies were photographed (top panels). Cells, scraped from colonies, were examined by microscopy to assess filamentation (bottom panels).
