# Supplementary material for: Mitochondrial proline catabolism activates Ras1/cAMP/PKA-induced filamentation in Candida albicans
Source: PLoS Genet. 2019 Feb 11;15(2):e1007976. doi: 10.1371/journal.pgen.1007976 (PMC6386415; doi:10.1371/journal.pgen.1007976)
Supplement: S4 Text — (DOCX) [file pgen.1007976.s011.docx]

**S4 Text**

**Supporting References**

106. Fonzi WA, Irwin MY. Isogenic strain construction and gene mapping in *Candida albicans*. Genetics. 1993;134(3):717-28. PubMed PMID: 8349105; PubMed Central PMCID: PMC1205510.

107. Wartenberg A, Linde J, Martin R, Schreiner M, Horn F, Jacobsen ID, et al. Microevolution of *Candida albicans* in macrophages restores filamentation in a nonfilamentous mutant. PLoS Genet. 2014;10(12):e1004824. doi: 10.1371/journal.pgen.1004824. PubMed PMID: 25474009; PubMed Central PMCID: PMC4256171.

108. Dabas N, Morschhauser J. Control of ammonium permease expression and filamentous growth by the GATA transcription factors GLN3 and GAT1 in Candida albicans. Eukaryot Cell. 2007;6(5):875-88. Epub 2007/03/21. doi: 10.1128/EC.00307-06. PubMed PMID: 17369441; PubMed Central PMCID: PMCPMC1899240.

109. Naglik JR, Rodgers CA, Shirlaw PJ, Dobbie JL, Fernandes-Naglik LL, Greenspan D, et al. Differential expression of *Candida albicans* secreted aspartyl proteinase and phospholipase B genes in humans correlates with active oral and vaginal infections. J Infect Dis. 2003;188(3):469-79. Epub 2003/07/19. doi: 10.1086/376536. PubMed PMID: 12870130.
